# Recent Advances: The Imbalance of Immune Cells and Cytokines in the Pathogenesis of Hepatocellular Carcinoma

**DOI:** 10.3390/diagnostics10050338

**Published:** 2020-05-25

**Authors:** Kumar Jayant, Nagy Habib, Kai W. Huang, Jane Warwick, Ramesh Arasaradnam

**Affiliations:** 1 Warwick Medical School, University of Warwick, Coventry CV4 7H, UK; j.warwick@warwick.ac.uk (J.W.); R.Arasaradnam@warwick.ac.uk (R.A.); 2 Department of Surgery and Cancer, Imperial College London, London SW7 5NH, UK; nagy.habib@imperial.ac.uk (N.H.); skyntuh@gmail.com (K.W.H.); 3 Department of Surgery & Hepatitis Research Center, National Taiwan University Hospital, Taipei 10002, Taiwan; 4 Centre of Mini-Invasive Interventional Oncology, National Taiwan University Hospital, Taipei 10002, Taiwan; 5 Graduate Institute of Clinical Medicine, College of Medicine, National Taiwan University, Taipei 10002, Taiwan

**Keywords:** hepatocellular carcinoma, immunomodulation, radiofrequency, check point inhibitors

## Abstract

Recent advancement in the immunological understanding of genesis of hepatocellular carcinoma (HCC) has implicated a decline in anti-tumour immunity on the background of chronic inflammatory state of liver parenchyma. The development of HCC involves a network of immunological activity in the tumour microenvironment involving continuous interaction between tumour and stromal cells. The reduction in anti-tumour immunity is secondary to changes in various immune cells and cytokines, and the tumour microenvironment plays a critical role in modulating the process of liver fibrosis, hepatocarcinogenesis, epithelial-mesenchymal transition (EMT), tumor invasion and metastasis. Thus, it is considered as one of primary factor behind the despicable tumour behavior and observed poor survival; along with increased risk of recurrence following treatment in HCC. The primary intent of the present review is to facilitate the understanding of the complex network of immunological interactions of various immune cells, cytokines and tumour cells associated with the development and progression of HCC.

## 1. Introduction

Hepatocellular carcinoma (HCC) has caused significant health problem, and according to the GLOBOCAN 2018 database, the annual incidence of the disease is almost 841,000 with 782,000 deaths. HCC is ranked sixth among the most commonly encountered malignancy and is the fourth most common cause of cancer-related mortality [1]. Despite the advancement of various surveillance systems and therapeutic strategies, the prognosis of HCC has remained dismal, and it continues to bring significant burden on healthcare delivery systems owing to late presentation, higher recurrence and metastasis. The prognosis of this cancer is directly dependent on the stage of cancer at presentation. During the early stage of presentation, hepatic resection or transplantation is considered as the therapeutic option of choice; however, the reported five-year survival rate is between 40% and 70% owing to increased recurrences and metastasis. Moreover, in advanced disease, prognosis is even worse with a median survival of between 3 and 11 months [2,3,4]. Contemporary studies have demonstrated that chronic inflammation and an associated decline in anti-tumour immunity create a conducive environment for the onset of HCC. The proof of concept provided another theory that an immune-mediated phenomenon can be modulated accordingly to treat the malignancy [5,6]. The therapeutic approach is based on synergism between radiofrequency (RF) energy and immune checkpoint inhibitors. In this review, we first outline a detailed overview to answer the following questions: ‘how does chronic inflammation in liver parenchyma modulate antitumor immunity?’ and ‘how does this intricate and heterogeneous landscape of immunological changes in liver parenchyma facilitate onset and growth of HCC?′ Based on the fact that specific immune compositions may extend tumour growth, an improved understanding of the functioning of immune cells and increase in knowledge of diverse mechanisms of immune evasion will help in modulating the anti-tumour immune response, we have highlighted the application of checkpoint inhibitors or with a combined approach of targeted immunotherapy and radiofrequency.

The precancerous state of HCC is marked by prolonged chronic inflammation of the liver, predominantly as a result of hepatitis B virus (HBV) and hepatitis C virus (HCV), other infections and non-alcoholic steatohepatitis [7,8,9]. The risk factors of HCC lead to a chronic inflammatory state, and the proclivities of proinflammatory cells present within the tumour microenvironment engender carcinogenesis and dysregulated growth [10,11,12]; studies have outlined that continued expression of cytokines and recruitment of immune cells on the background of a chronic inflammatory state might cause DNA damage and, thereby, genetic mutations and neoplastic transformation [13,14,15].

Often, the host immune system is diligent in detecting and eliminating such aberrant changes and prevents the occurrence of malignancy; nevertheless, studies have outlined similar observations in various cancers, including melanoma and renal cell carcinoma, where spontaneous regression of a tumour has been discerned alongside the marked clonal expansion of tumour-specific T-cells [16,17,18].

However, that is not always the case, and the background inflammatory state of liver parenchyma brings significant altercation in the tumour microenvironment in order to dodge the immune system, owing to alterations in molecular and cellular pathways involved in antigen processing, presentation and degradation of HCC cells [19,20,21]. These changes bring a paradigm shift in the immune response from anti-tumour to the state of tumour tolerance, leading to induction of the cancer process and progress of HCC. Thus, evasions in the natural anti-tumour immune response are essential prerequisites for the genesis of liver cancer. Liver cancer developing on the background of chronic inflammation and proinflammatory cells present in the tumour’s microenvironment not only influences the natural course of the disease but also determines the survival and recurrence following surgical resection [22,23,24].

## 2. Chronic Inflammation and Tumour Specific Immune Response in HCC

Mutations accrued within chronically inflamed hepatic parenchyma release loads of neo-antigens and are presented by dendritic cells (DCs) to trigger primary stimulus to initiate the immune response; however, the process requires additional co-stimulatory signalling to generate highly avid T cells to facilitate lysis of HCC cells. The upregulation of co-stimulatory activating molecules plays an important role in determining T cells’ response against tumour cells. The absence of anti-tumour immune response not only prevents tumour cell lysis but also inflicts apoptosis and anergy of the immune cells [22,25,26,27].

**Figure 1:**
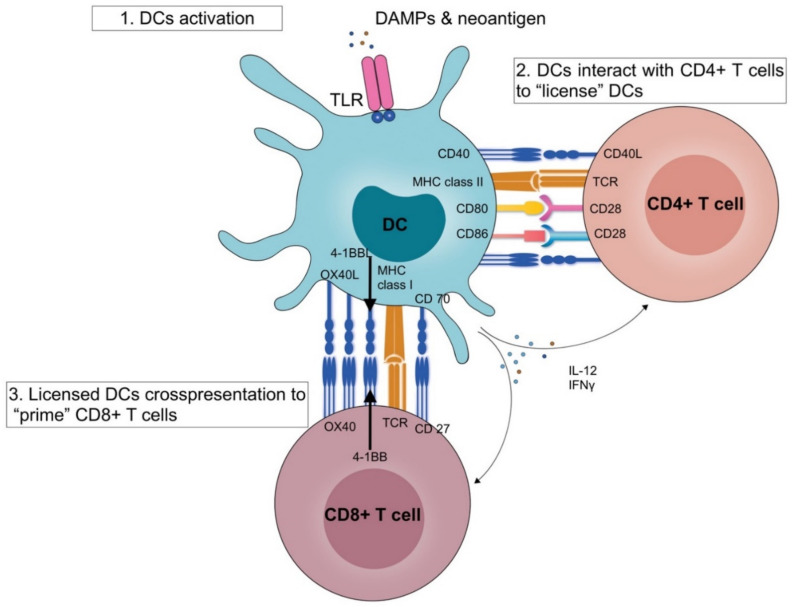
Pictorial depiction of activation of dendritic cells (DCs) by damage-associated molecular patterns (DAMPs) or neoantigens and subsequently enhanced expression of CD40, major histocompatibility complex (MHC) molecules and the co-stimulatory molecules CD80 and CD86. MHC II-mediated presentation of antigen to specific CD4+ T cells, with adjoining co-stimulatory signals (from CD80 and/or CD86) leads to activation of CD4+ T cells. The enhanced expression of the DCs’ licensing factors CD40 ligand (CD40L) and (LTα1β2) and binding between the CD40 and LTβ receptor (LTβR) to ‘licenses DCs′. Additionally, the process promotes release of IFNγ, IL-4, IL-12 and other cytokines, which in turn increase expression of CD70, CD86, 4-1BB ligand (4-1BBL), OX40 ligand (OX40L) and GITR ligand (GITRL). Priming of CD8+ T cells via MHC upregulation of CD27, 4-1BB, OX40 and GITR and binding with respective ligands bring further enhancement in CD8+ T cell functioning. Abbreviations—CD: cluster of differentiation; DAMPs: damage-associated molecular patterns; GITR: clucocorticoid-induced TNFR-related protein; GITRL: glucocorticoid-induced TNFR-related protein ligand; IL-12: interleukin 12; IFNγ: interferon γ; LTα1β2: lymphotoxin-α1β2; MHC: major histocompatibility complex; TLR: Toll-like receptor.

The DCs are most important antigen presenting cells (APC) and play a cardinal role in orchestrating the immune response in hepatic parenchyma [28,29]. The activation of DCs is essential in facilitating the binding between the recognition pattern receptors, Toll-like receptors (TLRs) and damage-associated molecular patterns (DAMPs) from tumour cells [30,31]. The pattern recognition receptors (TLR—Toll-like receptor) on DCs interact with DAMPs released by dysplastic cells of cirrhotic liver to acquire an activated form. Activated DCs promote myriads of cellular activities, including antigen processing thorough cross-presentation, expression of costimulatory molecules, release of inflammatory cytokines, migration to regional lymph node and presentation of processed antigens via major histocompatibility complex (MHC) class II molecules to naive CD4+ T cells [32,33] (Figure 1).

The interaction between MHC class II to T cell receptor (TCR) and CD80/CD86 with CD28 on naive CD4+ T cells induces primary and co-stimulatory signalling, thereby activation and proliferation of CD4+ T cells [34,35]. Further, the CD40 ligand-expressed over-activated CD4+ T cells bind with CD40 molecules on APCs. The interaction leads to the release of IL-12, which initiates the differentiation of T cells into type 1 T helper (Th1) cells also known as Th1 polarization and IFNγ production [36,37]. Thereupon, Th1 cells aid in cross-presentation of exogenous antigens via MHC class I molecules to CD8+ T cells to facilitate instigation into CD8+ cytotoxic T lymphocytes (CTLs) [38,39]. CTLs also induce secretion of IFNγ and release granzyme B and perforin to cytolyze HCC cells [40,41]. Hence, the anti-cancer immune response involves induction of the Th1 response, DC-medicated cross-presentation, activation of pro-inflammatory or tumour-associated macrophages (TAM), Tregs, myeloid-derived suppressor cells (MDSCs), NK cells and cytotoxic T cells to orchestrate optimum immune reaction against HCC cells [6,22,42]. Nevertheless, HCC frequently surfaces over an inflammatory background owing to failure in the anti-tumour immune response through induction of various immunosuppressive mechanisms. The tolerogenic property of liver helps in maintaining balance between beneficial immune responses against the acute exposure of virulent antigens in contrast to the perilousness of sustained immune response or chronic inflammation in the case of persistent exposure. Thus, evasions of the natural anti-tumour immune response are essential prerequisites for the genesis of liver cancer. The context of chronic inflammation and proinflammatory cells present in the microenvironment of HCC tumours is to influence the natural course of the disease and determine the survival and recurrence following surgical resection. During evaluation of the resectability of liver tumours, the following determinants are taken into account whilst contemplating the therapeutic approach: the extent of disease, size and location of tumour and underlying hepatic function, as the preservation of non-cancerous liver parenchyma is associated with reduced post-hepatectomy liver failure, decreased morbidity and mortality [43,44].

## 3. Neoantigens and Tumor-Associated Antigens in HCC

Hepatic cells accumulate cancer-specific neoantigens through genetic and epigenetic alterations with the intension of initiating the aberrant neoplastic growth [45,46]. Neoantigens are products of common mutations specific for certain malignancies taking place secondary to point mutations, gene amplifications, abnormal splicing and fusion genes, which maybe act as targets for CTLs. In contrast to other body cells, neoantigens are not introduced to the immune system as “self or non-self”, making them an ideal candidate to induce immune response. The malignant cells with higher antigenicity induce a relatively strong anti-tumour immune response and get eliminated; however, the tumour, which has developed in given circumstances, is more able to withstand the immune response [47,48,49,50].

Certain molecules have been identified to act as tumour-associated antigens (TAAs), cancer/testis (CT) antigens and differentiation antigens. Cancer Testis (CT) antigens, present in cancerous tissues including HCC, have resemblances to the peptides from the ovary, placenta and testis [51,52]. CT antigens do not have MHC; hence, the response of CTLs is selective to HCC cells bearing these antigens and does not affect normal tissue. Other antigens like melanoma antigen and CT antigen 1 are primarily noticed in melanoma and esophageal cancer; however, they can also be seen in HCC. Other TAAs that have been outlined in relation to HCC, including oncofetal protein observed in malignant cells, are also occasionally witnessed in normal tissues, such as α-fetoprotein and glypican-3 (GPC3) [53,54,55].

Owing to their ability to avoid thymic selection, TAAs can drive CTLs’ response in collaboration with CD4+ T cells to lyse tumour cells, and the degree of response is dependent on the frequency of mutations and likelihood of presenting T cell epitope [56,57]. Theories have linked TAA-specific CD8+ T cell immune response with reduced recurrence and improved survival [58]; however, the reality is deceived by the impaired IFNγ yield, as the efficacy of these responses seems to be misleading [59]. However, a recent study with sorafenib in advanced HCC demonstrated that enhanced expression of IFN-γ producing CD8+ T cells was associated with better progression-free survival and increased overall survival (OS). The increased frequencies of these effector T cells over Tregs were correlated with significantly reduced mortality risk and better overall survival [60].

## 4. Cell- and Cytokine-Mediated Changes in HCC Tumour Microenvironment

The development of HCC involves a network of immunological activity in the tumour microenvironment involving continuous interaction between the tumour and stromal cells. The tumour microenvironment plays a critical role in modulating the process of liver fibrosis, hepa- tocarcinogenesis, epithelial-mesenchymal transition (EMT), tumour invasion and metastasis.

The HCC microenvironment consists of (a) stromal cells, such as immune cells, carcinoma-associated fibroblasts (CAFs), hepatic stellate cells (HSCs) and endothelial cells (b) growth factors and inflammatory cytokines, and (c) extracellular matrix proteins [61,62,63]. One of the primary aims of this review is to facilitate understanding of the interaction between immune cells, cytokines and tumour cells.

## 5. Macrophages and Associated Cytokines

### 5.1. Tumour-Associated Macrophages and Associated Cytokines

Tumour-associated macrophages (TAMs) are mature macrophages arising from circulatory monocytic precursors and are one of the primary inflammatory cells involved with HCC-related inflammation. The phenotypic polarization of macrophages to the classical (M1) or alternative activation (M2) pathway occurs secondary to plasticity and is able to exercise both anti- and protumor activities respectively [36,64,65].

The classical activation pathway (M1) prevails in the presence of Th1 following response to microbial stimuli in the presence of cytokines, IFNγ and TLR ligands and results in release of additional inflammatory cytokines (IL-1b, IL-6, IL-12 and TNF-a) from macrophages, thereupon further enhancing Th1-mediated immune response and cytotoxic activity towards cancer cells, by producing high amounts of toxic intermediates, such as reactive oxygen species (ROS) and nitric oxide (NO) [64,66].

On the contrary, TAMs attain M2 phenotypes in the presence of cytokines such as interleukin 4 (IL-4), interleukin 13 (IL-13), transforming growth factor (TGF-β) and immune complexes/Toll-like receptor (TLR) ligands. The characteristics of index phenotypes include poor antigen-presenting capability, expression of a distinctive set of cytokines like IL-10 and transforming growth factor (TGF-β) and chemokines (CCL2, CCL22 and CCL 24), which suppress anti-tumour immune response to facilitate tumour growth, invasion and metastasis [67,68].

Studies have outlined that differentiation of macrophages into either M1 or M2 is dynamic and varies between/within HCC tumour nodules according to the predominant signal types, which is in turn determined by the tumour microenvironment. Thus, the polarization process of macrophages is determined by the tumour stage; the chronic inflammation helps in setting the M1 phenotype, whilst TAMs turn into M2 phenotypes in established HCCs [69,70]. However, contemporary research unraveled a “mixed” presentation of TAMs within different zones of HCC tumour nodules [59]. The plausible explanation of the above presentation could be related to the soluble mediators released by malignant cells within different regions of HCC nodules, which destines the polarization of macrophages by triggering transient early activation of monocytes in peritumoral stroma with high expression of HLA-DR (human leukocyte antigen), interleukin 1 beta (IL-1b) and inducing immunosuppressive M2 phenotypes in cancer nests [71,72,73].

Additionally, analysis of the state of the macrophage and involved molecules has exhibited their potential in deducing the HCC tumour behaviour and prognosis. The malignant hepatocytes express glypican-3 and secrete various molecules such as platelet-derived growth factor (PDGF), vascular endothelial growth factor (VEGF), TGF-β, CCL2 and MCSF to aid and abet the activation and recruitment of TAMs within the peritumoural region of HCC nodules [74]. A multitude of studies have demonstrated the positive correlation of enhanced expression of TAMs with angiogenesis, distant metastasis and poor prognosis of HCC [75,76,77].

### 5.2. Myeloid-Derived Suppressor Cells (MDSCs) and Associated Cytokines

Myeloid-derived suppressor cells (MDSCs) are a bone-marrow-derived heterogenous population of immature myeloid cells with various stages of differentiation. The distribution pattern involves bone marrow, peripheral blood, spleen and tumour mass, which inhibit the immune response against HCC cells. The proliferation and migration of MDSCs within the tumour microenvironment is mediated through granulocyte macrophage colony stimulating factor (GM-CSF), which is released by HCC cells [78,79]. The primary immunosuppressive mechanisms of MDSCs include the induction of Tregs and inhibition of T cells and NK cells. The notable molecules involved in MDSC-mediated immunosuppression are arginase (ARG1), inducible nitric oxide synthase (iNOS), IDO, ROS, TGF-β and IL-10 [80,81,82]. Furthermore, CD4+ and CD8+ T cell mediated immune response is constrained through depleting arginine following induction of arginase, release of ROS and NO and downregulating of L-selectin [83]. Exhausted stores of l-arginine and increased levels of NO and ROS result in the suppression or desensitization of the T-cell receptor or anergy induction of T-cell [84]. In addition, IDO-mediated breakdown of L-tryptophan downregulates T and NK cells and induces Tregs [85]. Moreover, MDSCs secrete various angiogenic factors and vascular-modulating enzymes to stimulate angiogenesis through granulocyte colony-stimulating factor (G-CSF)-dependent STAT3 signaling, thus rendering refractoriness to the conventional anti-VEGF therapy. However, this also offers opportunities for clinical development of drugs, which can interrupt the infiltration or functioning of MDSCs with the intension of reestablishing or boosting the effect of anti-VEGF drugs [86].

In addition, the interaction between MDSCs and TAMs helps in maintaining balance among Th1/Th2 and M1/M2. The downregulation of IL-12 production from TAMs is caused by release of IL-10 from MDSCs, which in turn leads to Th2 polarization [87]. Furthermore, release of IL-4 from Th2 precipitates the activation of the alternative pathway for macrophage maturation (M2) causing further decline in the anti-tumour immune response [64,88].

Indoleamine 2,3-dioxygenase (IDO) is involved in the cellular metabolism of tryptophan and acknowledged for its role in progression of cancer, facilitating immune tolerance, induction of apoptosis in T-cells via tryptophan depletion and enhancing differentiation of CD4 T-cells to Tregs [89,90,91]. Studies demonstrated that a higher expression of IDO in tumour nodules is associated with poorer prognosis [92]. Based on these finding, IDO inhibitors, indoximod and epacadostat, have been developed and preliminary data demonstrates their potential in stabilizing tumours; however, further studies are required to elucidate the potential in terms of anti-tumour immune responses and their applicability with checkpoint inhibitors, which will determined following completion of an ongoing clinical trial [93].

In light of the above observation, the protumor effects of MDSCs could be reversed via various means such as depletion of MDSCs, creating hindrance in MDSC trafficking and migration into TME and inhibition of the immunosuppressive properties of MDSCs.

### 5.3. Dendritic Cells and Associated Cytokines

**Figure 2:**
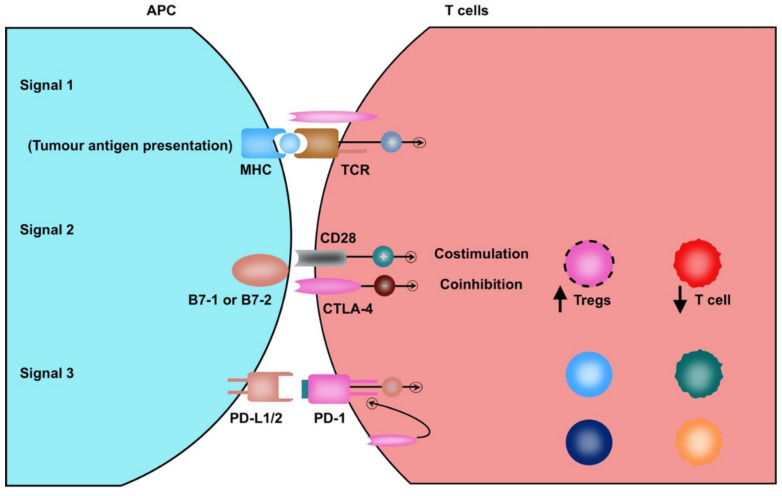
Pictorial depiction of T-cell activation and modulation of T-cell functioning in hepatocellular carcinoma (HCC). TCR (T-cell receptor) expressed on surface of T cells recognizes a tumour antigen through MHC I/II of APC (antigen presenting cells) (signal 1). The sensitivity of antigen recognition by TCRs is further enhanced by CD4 and CD8 coreceptors. T cell effector function is gained through costimulatory receptors (signal 2). The interaction between CD28–B7-1/B7-2 initiates a co-activator signal, whilst interplay involving CTLA-4–B7-1/B7-2 inhibits T-cell activation. Further, CD28 and CTLA-4 play a critical role in the development and function of Tregs. The inflammatory state of hepatic parenchyma causes increased expression of PD-1. Further PD-1–PD-L1 interaction (signal 3) is involved in the inhibition of T-cell activity and increase in Tregs.

The DCs are the most efficient APCs and essential in establishing the anti-tumour immune response [94]. The pattern recognition receptors (TLR—Toll-like receptor) on DCs interact with DAMPs released from dysplastic cells of cirrhotic liver to attain an activated form. In accordance with the earlier description, the process of classical cross-priming involves presentation of tumour-derived neoantigens by MHC class II on DCs to CD4+ T helper cells, which in turn ‘license′ the DCs through CD40 ligand (CD40L) and CD40 interactions for cross-priming and facilitate the release of cytokines IL-12 and IFNγ [33] (Figure 1). The licensed DCs migrate to regional lymph nodes and upregulate the expression of various co-stimulatory molecules, including CD80, CD86, CD70, OX40L, GITRL (glucocorticoid-induced TNFR-related protein ligand) and 4-1BBL for cross-presentation to CD8+ T cells, along with downregulate the expression of inhibitory molecules, programmed cell death ligand (PD-1) and CTLA-4 [95]. The coupling of respective molecules results in robust CD8+ T cell activation, proliferation and effector function [96] (Figure 2).

On the flip side, the immune checkpoint molecules CTLA4 and PD-1 are inhibitory receptors expressed upon activated T cells, which bind with respective ligands expressed over DCs and MDSC and HCC cells [97,98]; the interaction not only suppresses cytotoxic activity of CD8+ T cells but also promotes tumour growth through increased signaling of the mammalian target of rapamycin (mTOR) independent of PI3K [99,100].

### 5.4. T Lymphocytes and Associated Cytokines

Both types of T lymphocytes CD4+ T helper cells and CD8+ cytotoxic T cells ensure the anti-tumour immune response through inhibiting the occurrence and proliferation of HCC [101]. The activation of naive CD4+ T cells by DCs not only releases cytokines (IL-4, IL-12 and IFNγ) within the tumour microenvironment but also facilitates its own (CD4+ T cells) differentiation into Th1 cells [102]. Furthermore, autoinduction of the secretion of pro-inflammatory cytokines as IFNγ enhances expression of MHCII to facilitate direct recognition by CD4+ T cells and aid in the activation of CD8+ T cells and macrophages. Additionally, IL-4 induces the transformation of CD4+ T cells into Th2 cells, initiates production of IL-4, IL-5 and IL-13 and underpins the humoral immune response [103,104]. Moreover, macrophage colony-stimulating factor (M-CSF) and chemokine-CC motif-Ligand2 (CCL2), present in the tumour microenvironment, facilitate interaction between HCC cells and stromal components and enhance cellular infiltration [105].

Contemporary evidence suggests that Th1 cytokines are directly reciprocal to improved survival in HCCs, whilst heightened levels of Th2 cytokines are indicative of vascular invasion and recurrences [106]. Reversal of protumour activity of T lymphocytes could be achieved by the development of a methodology to suppport the transformation of CD4+ T cells toward Th1 cells, hence reinforcing the activation and infiltration of CD8+ T cells into the tumour microenvironment of HCC [107].

### 5.5. Regulatory T Cells and Associated Cytokines

Regulatory T cells (Tregs) are a subtype of CD4+ T cells, which express CD25 (IL-2 receptor) on the surface with intracellular transcription factor forkhead box P3 (FoxP3) [108]. They are of paramount importance in immunosuppression as a regulator of self-reactive T cells and promoter of peripheral immune tolerance. The co-presence of TGF-β and IL-2 instigates the expression of FoxP3 and incites differentiation of naive CD4+ T cells to Tregs, alongside increased expression of IL-2 receptors causing decline in IL-2 levels. IL-2 plays an important role in the differentiation and proliferation of CTLs, hence reduction in IL-2 further exacerbates immunosuppression [109].

Additionally, Tregs breakdown extracellular ATP to adenosine, which interacts with reciprocal receptors (CD39 and CD73) present on effector T cells to suppress their activity. Moreover, Tregs are also implicated in expression of various other regulatory cytokines, chemokines and chemicals: TGF-β and IL-15 cause suppression of T-cell proliferation; CTLA-4, which binds with CD80/86 on T cells to dampen their functioning; Granzyme B leads to apoptosis of effector T cells; epidermal growth factors mediate proliferation of epithelial cells and extracellular matrix; downregulating of NK group 2 member D expression and dampening of NK cell activity; and chemokine C-C motif ligand 20 assisted migration of Tregs to HCC nodules [110,111]. Hence, to recapitulate, Tregs not only establishes an immunosuppressive microenvironment to foster HCC growth but also acts as a marker of poor prognosis [112]. A recent study has shown that anti-CTLA-4 antibodies (Ipilimumab) act through the interaction of CTLA-4 on Tregs and effector T cells thereupon causing depletion of Tregs by limiting the negative signaling and immunosuppressive function [113].

### 5.6. NK Cells and Associated Cytokines

NK cells are a member of the innate immune system and have a cytotoxic effect equivalent to CTLs of the adaptive immune system. In addition, they secrete several cytokines to regulate the activity of other immune cells [114,115]. One of the most important attributes of NK cells is their ability to modulate functioning in accordance with the tissue microenvironment. Herein, hepatic NK cells are higher in proportion (30–50%) and more aggressive in terms of cytotoxicity and cytokine production than peripheral NK cells. NK cells are divided into two subtypes based on degree of expression of CD56 as CD56 bright and CD56 dim. The precursor NK cells differentiate into CD56 bright NK cells, which in turn give rise to the dim NK cells (almost 90% of peripheral NK cells and 50% of hepatic NK cells). The CD56 dim NK cells are more cytotoxic towards target cells but secrete less cytokines than bright NK cells [116,117]. Studies have demonstrated reduced levels of peripheral and hepatic NK cells in HCC patients in contrast to normal healthy individuals [118,119,120]. Several schools of thought have been put forward in attempt to elucidate the nature of NK cell functions in connection with cirrhosis and HCC. A decline in the CD56 dim NK are observed in circulating blood and in HCC tumour nodules, which suggests that the suppressed tumour-surveillance functions of NK cells is caused by a decline in the release of IFNγ and cytotoxic activity in HCC patients. The dysfunction of NK cells in HCC is mediated by monocytes/macrophages and fibroblasts through NKp30 receptor, MDSCs, CD48/2B4 interactions and IDO, respectively [118,119,120].

In contrast to T cells, NK cells can engender immune response more expeditiously and do not depend on MHC. This trait is of paramount importance against malignant cells that lack MHC I molecules, which can still be recognized and culled by NK cells. The merits of NK cell-mediated immune response are inherent to the mechanism of how they target MHC I missing cancer cells. NK cells function according to the balance in the signals from killer activation receptors (KARs) and inhibitory receptors present over these cells. Natural killer group 2D (NKG2D) belongs to KARs group of inhibitory receptors and includes inhibitory killer-cell immunoglobulin-like receptors (KIRs) and receptors called immune checkpoint molecules including PD-1 and T-cell immunoglobulin and mucin-domain containing-3 (TIM3). NK cells use KIRs to detect the target cells and assess the level of expression of MHC I. An insufficient expression of MHC I molecules leads to tenuous engagement with KIRs and proceeds with killing of the target malignant cells; however, adequate participation of MHC I molecules to KIRs forestalls target cell killing as the signal to destroy is rescinded by the suppression signal. Additionally, DNA damage and cell stress in target cells upregulates NKG2D ligand [121,122,123,124]. In contrast, persistent revelation of the NKG2D ligand to NK cells can downregulate NKG2D and inflict anergy into them [125,126].

Considering the above observation, reinstatement NK cell functioning might be influential in HCC immune therapy. This could be achieved through either endogenous stimulation of the NK cells or adoptive NK cell therapy. Alongside this, reinstitution of NK cells might enhance the therapeutic efficacy of sorafenib and anti-programmed death-ligand 1 (PD-L1) monoclonal antibody and bring significant reduction of HCC tumour nodules [114,127]. However, further studies are required to understand the clinical efficacy and toxicity profile of these drugs.

### 5.7. Hepatic Stellate Cells, Endothelial Cells, and Cancer-Associated Fibroblasts

It has been proposed that the premalignant microenvironment and tumour microenvironment in HCC should be differentiated. At the early stages it is characterized by chronic liver injury, inflammation and fibrosis and as preceding tumour formation, whereas later it evolves in the already developed tumour [128]. Liver fibrosis is a protective response to heal the acute insult to the liver parenchyma; however, the persistence of inciting factors turns fibrosis into cirrhosis and dysfunctional hepatic tissue. Ongoing insults modulate hepatic fibrogenesis by facilitating activation of hepatic stellate cells (HSCs) into myofibroblasts, recruitment of inflammatory cells, migration of alpha-smooth muscle actin, secretion of cytokines and chemokines and accumulation of extracellular matrix (ECM) components at the injury site. Moreover, the inflammatory cells, activated macrophages and HSCs trigger production of ROS that not only encourage fibrosis through activation and migration of HSCs, but can also incur neoplastic changes by undesirable injury and mutations in hepatocytes, or by deterring tumour immunosurveillance [129,130]. Consequently, it has been reported that antioxidants can effectively reduce hepatocarcinogenesis through inhibition of ROS [131].

HSCs and endothelial cells release the C-X-C motif chemokine 12 (CXCL12) or stromal cell-derived factor 1. CXCL12 is involved in induction tumour growth, migration and invasiveness via engagement of C-X-C chemokine receptor type 4 (CXCR4) on malignant cells and also promotes tumour angiogenesis through mediation of endothelial progenitor molecules [132,133]. Additionally, the interaction of CXCL12 with CXCR4 recruits MDSCs, and endothelial cells induce Tregs through TGF-β; as mentioned earlier, both are implicated in the generation of the immunosuppressive tumour microenvironment [80,81]. Furthermore, increased expression of FasL over these cells also aids in elimination of infiltrating CTLs and increases the risk of tumour invasion and metastasis [110,111,134,135].

## 6. Future Implications

**Figure 3:**
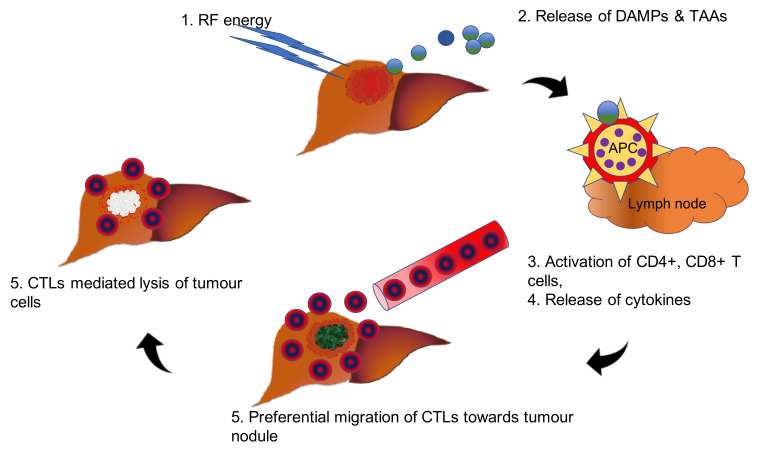
Schematic representation of release of DAMPs following radiofrequency-mediated ablation HCC nodules and thereby increased influx of cytotoxic T lymphocytes and reinstatement of the antitumour immune response. Abbreviations—APC: antigen presenting cells; CTLs: cytotoxic T lymphocytes; RF: radiofrequency.

Normally, the host immune system perceives and eliminates any aberrant premalignant changes owing to the chronic inflammatory state of liver parenchyma and limits the emergence of an oncological dilemma, i.e., HCC. This fact is supported by the reports of spontaneous regression in various cancers, including melanoma and renal cell carcinoma, which suggest that the marked clonal expansion of tumour-specific T cells in protection against malignancy [16,17,18]. Moreover, this theory is further supported by the ‘abscopal effect′ described in literature in relation to various solid tumours such as melanoma, renal cell carcinoma and HCC [136]. Recent studies have outlined local therapy such radiofrequency (RF) in modulation of tumour-mediated host immune response. The application of RF energy over HCC nodules not only kills the tumour cells but also releases an abundance of neoantigens and DAMPs. The release of DAMPs results in increase in serum lympohocytes and incites CD4+ and CTLs and NK cells in tumour parenchyma, which shift the scale of balance towards an anti-tumour immune response rather from an tumour suppressive state [137] (Figure 3).

The anti-tumour positive immunomodulatory change is more discerned in terms of Tregs, CD8+ T cells, TGF-β, IFNγ, IL-10 and IL-17 respectively [138,139,140,141,142]. Furthermore, increased expression of checkpoints such as PD-L1/PD-L2/PD-1 (programmed death ligand 1 or 2/programmed cell death 1 receptor) and CTLA-4 have been implicated in the inhibition of immune activity in the hepatic milieu, particularly in instances of chronic inflammation of liver [143,144]. The applicability of the combination of various checkpoint inhibitors like anti-CTLA or anti-PD-1 drugs with other ablative therapies such as radiofrequency to further enhance the antitumour immune response and survival has been discussed. Both rationales call for combining these two modalities. The proposition of involving radiofrequency ablation with checkpoint inhibitors seems a pragmatic approach to invigorate an antitumour immune response against HCC cells [145]. However, further studies are required to understand their applicability in the early stages of HCC, a classical archetype of inflammation-associated malignancy, since most tumours arise in the context of hepatic inflammation and the resultant fibrosis.
